# Factors associated with caregivers’ consistency of use of bed nets in Nigeria: a multilevel multinomial analysis of survey data

**DOI:** 10.1186/s12936-018-2427-x

**Published:** 2018-08-02

**Authors:** Stella Babalola, Sulaimon T. Adedokun, Anna McCartney-Melstad, Mathew Okoh, Sola Asa, Ian Tweedie, Andrew Tompsett

**Affiliations:** 1 Breakthrough Action/Johns Hopkins Center for Communication Programs, Johns Hopkins University, 111 Market Place – Suite 310, Baltimore, MD 21202 USA; 2 Department of Demography and Social Statistics, Obafemi Awolowo University, Ile-Ife, Nigeria; 3 Breakthrough Action/Johns Hopkins Center for Communication Programs, Abuja, Nigeria; 4 USAID/President’s Malaria Initiative, Washington, DC USA

**Keywords:** Net use, Ideation, Multilevel, Nigeria

## Abstract

**Background:**

Malaria remains endemic in Nigeria despite programmes and policies put in place toward malaria elimination. Long-lasting insecticidal nets have been documented to offer protection from malaria by preventing mosquito bites. While many studies have examined the factors associated with the use of bed nets in Nigeria and across Africa, little information is available on the factors associated with consistency of use of bed nets.

**Methods:**

The data for this study were derived from a household survey conducted in three states in Nigeria (Akwa Ibom, Kebbi and Nasarawa) between July and September 2015 by the Health Communication Capacity Collaborative, a 5-year cooperative agreement supported by the United States Agency for International Development and the US President’s Malaria initiative and led by the Johns Hopkins Center for Communication Programs. The analysis was limited to a total of 3884 men and women selected from 2863 households with at least one bed net. Multilevel multinomial logistic regression was used to assess the factors associated with consistency of use of bed nets.

**Results:**

The findings revealed 43.2% of the respondents use bed nets every night, while 38.4% use bed nets most nights. The factors associated with using a bed net every night rather than rarely or never using a bed net included sociodemographic and household variables (age, gender, religion, household size, net density, and household wealth), ideational variables (perceptions about severity, susceptibility, self-efficacy to use nets, and response-efficacy of bed net; awareness of place of purchase; willingness to pay for bed nets; attitudes towards net use; and descriptive norm about nets), and state of residence. The three study states differ significantly in terms of most of the independent variables included in the estimated model.

**Conclusions:**

The study recommends that efforts designed to promote consistent use of bed nets should be state-specific and include strategies targeting ideational variables. Furthermore, given the significance of unmeasured heterogeneity at the cluster level, strategies to engage and mobilize the community, such as community dialogue, home visits and engaging community leadership, are relevant.

## Background

Malaria is endemic to Nigeria and the majority of the population is at risk. Indeed, Nigeria accounted for 52% of the 110 million malaria cases in the West Africa region in 2016 [1]. Insecticide-treated nets (ITNs; including long-lasting insecticidal nets—LLIN) represent the most cost-effective malaria prevention strategy. ITNs not only prevent physical human and mosquito contact, but also repel and/or kill mosquitoes [2–4]. WHO recognizes this strategy as the primary prevention method and recommends mass distribution campaigns to scale up ITNs in areas prone to malaria [1]. Use of LLINs, remains the cornerstone of malaria prevention in Nigeria [5].

Nigeria’s 2014–2020 National Malaria Strategic Plan aims to reduce malaria burden to pre-elimination levels and reduce malaria-related mortality to zero by 2020 [6]. A key strategy to achieve the goals of the strategic plan is to expand universal access to LLINs. Between May 2013 and March 2015, with support from its strategic partners, the National Malaria Elimination Programme (NMEP) organized the distribution of 46 million LLINs nationwide. Whereas use of LLINs—defined as sleeping under a LLIN on the night preceding the survey—has increased considerably in recent years in Nigeria, net use has not yet become a consistent social norm across the country [5]. Findings from the 2015 Malaria Indicator Survey showed that while 69% of households own at least one LLIN (increasing from 42% in 2010), only 50% of household members slept under an LLIN on the night prior to the survey in households with at least one LLIN. Although the LLIN use to access ratio—the proportion of the population that slept under an LLIN the night before the survey divided by the proportion of the population with access to an LLIN within their household—has increased significantly in Nigeria in recent years, the indicator remains lower in the southern states compared to the northern ones and lower than in most other countries of the sub-region [7].

Most studies that have assessed the determinants of bed net use utilize the commonly used global indicator that measures net use on the night before the survey. Evidence from these studies indicates that having access to a bed net is a critical factor for net use [8–10]. In addition to access, factors that have been found to be associated with bed net use include age, gender, pregnancy status, household size, household sleeping arrangements, number of nets in the household, and socioeconomic status. Studies that have assessed the role of age have found that older children and teenagers are less likely than other household members to use bed nets; use is generally most common among children aged less than 5 years [11–14]. Some studies have found that adult women are more likely than their male peers to sleep under a net [15–18], and other studies have found that pregnant women are more likely than non-pregnant women to sleep under a bed net [11, 19].

Studies have documented that household sleeping arrangements, such as whether the person sleeps on a bed versus a mat or whether a child sleeps with the mother, influence the use of bed nets [13, 17, 18, 20]. Evidence from these studies suggests that sleeping on a bed is associated with increased bed net use compared to sleeping on a mat or any other object placed directly on the floor/ground, and that young children who sleep with their mother are more likely than other children to sleep under a net. Education of the head of household or the individual respondent has also been found to be correlated with increased net use in some studies [21, 22] and with decreased use in others [23]. However, the evidence regarding the role of household socioeconomic status largely suggests a positive relationship [17, 24].

Existing literature provides consistent evidence that net use is positively correlated with the number of nets in the household [17, 22, 25] and is negatively associated with household size [12, 22]. Similarly, studies have generally found higher net density—defined as number of nets per person in the household—to be positively associated with net use [15, 26]. For its part, urban residence has been found to be associated with increased net use in some studies [15, 16] but with decreased use in others [21].

In addition to sociodemographic and household characteristics, a few studies have examined the role of ideational (psychosocial) variables and community characteristics. The ideational determinants measured in these studies were knowledge about malaria and bed nets, descriptive norms about net ownership and use, perceived self-efficacy to use nets properly, perceived self-efficacy to detect signs of complicated malaria, perceived response efficacy of bed nets, perceived disadvantages of bed nets, perceived severity of malaria, and perceived susceptibility to malaria [10, 12, 21, 23, 25, 27, 28]. Furthermore, a few studies that examined the role of exposure to malaria prevention information in mass media have generally found a positive relationship with bed net use [10, 12, 29].

For effective prevention of malaria, it is important to use bed nets consistently, that is, every night. Research has demonstrated that consistent use of insecticide-treated bed nets can reduce the incidence of malaria by up to 90% and under-5 mortality by more than 40% [3, 4, 30]. While the inconsistent use of nets has been shown to limit the protective benefits of nets, only a few studies have examined the determinants of consistency of bed net use [18, 26]. This study is an attempt to bridge the knowledge gap. Using data collected from a household sample survey conducted in Nigeria in 2015, this study examines correlates of consistency of use of bed nets in three study states: Akwa Ibom, Kebbi and Nasarawa. While malaria is endemic to the three states, the states nonetheless differ in terms of malaria prevalence. For example, secondary analysis of the Malaria Indicator Survey (MIS) conducted by the lead author reveals significant differences in malaria prevalence among under-5 children: 22.70% in Akwa Ibom, 35.85% in Nasarawa, and 63.51% in Kebbi.

## Methods

### Setting

The data for this study were drawn from household sample survey data collected in Nigeria between July and October 2015 by the Health Communication Capacity Collaborative (HC3), a 5-year cooperative agreement supported by USAID and the US President’s Malaria initiative (PMI) and led by the Johns Hopkins Center for Communication Programs. The data were collected during the rainy season in three Nigerian States: Akwa Ibom, Kebbi, and Nasarawa. The three states represent three of the six geopolitical zones in Nigeria: South–South, North-West, and North-Central, respectively. While malaria is endemic to the three states, each state differs in terms of their social and health indicators. For example, secondary analysis of the 2013 Nigeria Demographic and Health Survey data (unpublished research by the lead author) showed that the proportion of women with post-primary education was 10.5% in Kebbi State, 42.4% in Nasarawa, and 71.6% in Akwa Ibom. The secondary analysis underscored the differences in ethnic diversity among the three states: the dominant ethnic group(s) in Kebbi State is the Hausa; in Akwa Ibom, are the Ibibio, Annang, and Oron; and in Nasarawa are the Eggon, Afa, Alago, Mada, and Koro.

### Design and procedure

The data analysed in this manuscript came from a larger survey designed to provide baseline data for the HC3 Malaria Project in Nigeria [31]. A multistage random sampling technique was employed to select respondents for the original survey. In the first stage, the local government areas (LGA) in each state were divided into two categories depending on whether or not the LGA was scheduled to receive HC3-implemented community-based activities. These two categories served as the survey strata in each state. Within each survey stratum, three LGAs were selected with probability proportional to size (PPS) for a total of six LGAs per state. Subsequently, 10 clusters (enumeration areas) were randomly selected from each LGA, for a total of 60 survey clusters per state. In each cluster, all the eligible households were listed and 20 randomly selected for participation in the survey. A household was eligible for participation if there was at least one child aged less than 5 years old in the household at the time of the survey. The mother of such a child was eligible to be interviewed if she was aged between 18 and 49 years at the time of the survey. In one-third of the selected households, the male head of household aged between 18 and 59 years and the father of a child aged less than 5 years old was also interviewed. From these households, a total of 3611 women and 1268 men consented to being interviewed. In all, relevant information about living conditions and net ownership and use was collected from 3616 households using a structured household survey instrument. More information about the sampling design is available elsewhere [31]. The analysis of the determinants of bed net use reported in this manuscript were limited to 3884 male and female caregivers selected from 2863 households with at least one bed net.

### Variables

In this study, the dependent variable was the consistency of use of bed nets, defined as a categorical variable with three possible values: sleep under a net every night (consistent use), most or some nights (6 nights a week or fewer; inconsistent use), and rarely or never. The analysis involved assessing the predictive value of the following independent variables:

i. Sociodemographic characteristics: gender, current age, level of education, and religion.
ii. Media habits (regular radio listenership, regular television viewership) and exposure to messages, specifically exposure to malaria related-information on media or through community sources.
iii. Ideational variables:
  1. Perceived severity of malaria
  2. Perceived susceptibility to malaria
  3. Positive attitude towards use of bed nets
  4. Perceived self-efficacy to prevent malaria
  5. Perceived self-efficacy to use bed nets
  6. Perceived response efficacy of bed nets
  7. Knowledge that malaria is caused by mosquito bites
  8. Perceived self-efficacy to detect severe malaria
  9. Knowledge of where to procure nets
  10. Discussion of malaria with others in last 12 months
  11. Discussion of bed nets with others
  12. Willingness to pay for bed nets
  13. Descriptive norm about bed nets (perceived net use to be a community norm)
iv. Household variables: household wealth quintile (an asset-based construct), household size, number of nets in the household.
v. Community variables: type of place of residence, state of residence, and composite score to assess community advantage.

Each of the first six ideational variables was derived from a set of Likert-scale items. Responses to the items were scored between − 2 and + 2, combined and then split at the median to denote higher and lower levels. Perceived self-efficacy to detect severe malaria was derived from a single Likert-scale question, while the descriptive norm variable was derived from a question that asked what proportion of families in the community of residence use bed nets. The other variables were derived from simple yes/no questions.

The composite score to assess community advantage was derived from four community compositional factors: prevalence of post-secondary education among women in the cluster of residence, prevalence of regular television viewership, proportion of non-poor households in the cluster, and prevalence of finished floor in the cluster. The score was derived using principal component analysis and split into three equal parts (tertiles).

### Analyses

The main analytic method used in this manuscript was multilevel multinomial logistic regression. Only significant factors identified at the bivariate level were included in the multinomial model. The model was estimated using generalized structural equation modeling (GSEM) using the *gsem* command in Stata version 14. The GSEM model assessed the fixed effects of various sociodemographic, ideational, household, and community variables. In addition, the model assessed random effects at the cluster level.

## Results

### Background characteristics of the study population

The background characteristics of the respondents are presented in Table 1. The study sample was comprised of 3884 respondents (73.8% women and 26.2% men) selected from 2863 households with at least one bed net and at least a child under 5 years old. Although the original sample included an almost equal proportion of respondents from each of the three states, among the households with at least one net, there were proportionally more respondents from Akwa Ibom (40.4%) compared to Kebbi (33.3%) and Nassarawa (26.4%). The modal age group was 25–34 years, very few respondents were older than 44 years. About 40.0% of the respondents had at least a secondary education while about one-third had no education. More than half (54.8%) of the respondents were Christians, and more respondents were in each of the highest two quintiles compared to the lowest two. This finding suggests that households with nets were richer than their peers without nets. About six out of every ten respondents were rural residents, and the overall proportion that listened to the radio regularly or watched the television regularly was 54.5 and 39.0%, respectively. The data presented in Table 1 furthers highlight significant differences in socio-demographic and household characteristics across states. Indeed, with the exception of gender, the three states differ significantly according to all the variables examined. For example, the Akwa Ibom sample includes proportionally more respondents in the 25–34 years or 35–44 years age categories and significantly fewer respondents in the youngest age categories compared to Kebbi or Nasarawa. Similarly, the respondents from Akwa Ibom are, on average, better educated than their peers from Kebbi or Nasarawa: about 5% of the respondents from Akwa Ibom had no formal education compared to 79% from Kebbi and 29% from Nasarawa. Whereas the Nasarawa sample includes equal proportions of Christians and Muslim, the Akwa Ibom sample is predominantly Christian and the Kebbi sample predominantly Muslim. Furthermore, the Akwa Ibom sample includes proportionally more respondents in the higher wealth quintiles than the Kebbi or Nasarawa sample. In addition, both regular radio listening and regular television viewing are more common in Akwa Ibom than in the other two states. These habits are least common in Kebbi state. Similarly, exposure to media messages on malaria prevention and treatment is most common in Akwa Ibom and least common in Kebbi.

**Table 1:** Percent distribution of respondents from households with at least one net, by socio-demographic, ideational and household characteristics; Nigeria 2015

Background characteristics	State			
	Akwa Ibom (n = 1568)	Kebbi (n = 1292)	Nasarawa (n = 1024)	All States (n = 3884)
Age group***				
18–24	17.28	26.78	22.46	21.81
25–34	48.53	41.02	46.88	40.60
35–44	24.62	21.52	21.97	22.89
45 +	9.57	10.68	8.69	9.71
Respondent’s sex				
Male	25.89	26.55	26.37	26.24
Female	74.11	73.45	73.63	73.76
Education level***				
None	5.17	78.95	29.20	36.05
Primary	32.46	7.97	31.64	24.10
Secondary/higher	62.37	13.08	39.16	39.85
Religion***				
Christian	99.17	4.18	50.78	54.81
Muslim	0.19	92.49	49.22	43.82
Others	0.64	3.33	0.00	1.36
Wealth index***				
Lowest	1.08	46.98	9.38	18.54
Second	6.19	25.46	23.44	17.15
Middle	20.03	12.77	28.42	19.82
Fourth	34.31	7.28	22.27	22.14
Highest	38.39	7.51	16.50	22.35
Place of residence***				
Rural	83.55	81.11	69.34	78.99
Urban	16.45	18.89	30.66	21.01
Listened to radio at least once a week***	77.04	28.56	52.64	54.48
Watched the television at least once a week***	61.67	18.03	33.01	39.60
Exposed to messages on malaria prevention from the media in last 12 months***	72.90	20.90	38.28	46.47
Perceived severity of malaria***	26.85	28.95	37.21	30.38
Perceived susceptibility to malaria***	53.38	28.72	38.28	41.19
Perceived self-efficacy to recognize symptoms of severe malaria***	74.81	78.87	59.08	72.07
Knowledge that malaria is caused by mosquitoes***	85.78	90.40	95.90	89.98
Perceived self-efficacy to use nets***	48.34	68.65	52.64	56.23
Discussed malaria with others in last 12 months***	94.07	79.18	82.32	86.02
Discussed nets with someone in last 12 months***	28.25	26.08	39.75	30.56
Perceived that use of bed net was a community norm***	66.26	61.30	51.56	60.74
Scored high (above median) for positive attitudes towards bed nets***	52.42	33.05	46.97	44.54
Higher level of perceived self-efficacy to prevent malaria***	67.54	76.56	66.60	70.39
Higher level of perceived response-efficacy of nets***	45.66	23.84	46.97	38.75
Knew where to purchase a net in their community***	13.07	46.59	20.41	26.16
Willing to pay for bed nets***	27.01	67.96	61.33	49.69

Significance of differences across states: ***p < *0.001*

Overall, the ideational characteristics of the respondents were such that less than one-third perceived malaria to be serious while only 41.1% perceived themselves to be susceptible to malaria. Similarly, only 38.7% believed in the efficacy of nets to prevent malaria and about a quarter (26.2%) knew of a place to purchase nets in their community. About half of the respondents were willing to pay for nets. In contrast, most of the respondents were confident in their ability to prevent malaria through any means (70.4%) and had discussed malaria with someone in the past 6 months (86.0%). As observed with the socio-demographic and household variables, the states differ conspicuously by ideational variables. For example, perceived severity of malaria is lower in Akwa Ibom than in Kebbi and Nasarawa whereas perceived susceptibility of malaria is higher in Akwa Ibom than in the other two states. The respondents from Kebbi are more likely than their peers from Akwa Ibom or Nassarawa to report perceived self-efficacy to use bed nets. The perception that bed net use is the norm in the community is less common in Nasarawa than in the other two states whereas positive attitudes towards bed nets are less prevalent in Kebbi than in the other two states. In contrast, the respondents from Kebbi are more likely than their peers in the other two states to perceive the self-efficacy for preventing malaria and to know a place in their community where bed nets can be purchased. Willingness to purchase nets is also more common in Kebbi state than in the other two states. Finally, the Akwa Ibom sample displays the lowest levels of awareness of a place to purchase nets and willingness to purchase nets.

### Use of bed nets: patterns and variations

Overall, 79.2% of the surveyed households had at least one bed net. Ownership of nets was more common in Akwa Ibom (96.5% of households) compared to Kebbi (78.9%) and Nasarawa (62.1%). The proportion of households with access—with at least one net for every two household members—was 30.4% in Akwa Ibom, 17.6% in Kebbi, and 15.3% in Nasarawa. In households with at least one bed net, 43.2% of respondents reported consistent bed net use (that is, every night) while 38.4% inconsistent use and 18.4% rarely or never used a bed net.

**Fig. 1:**
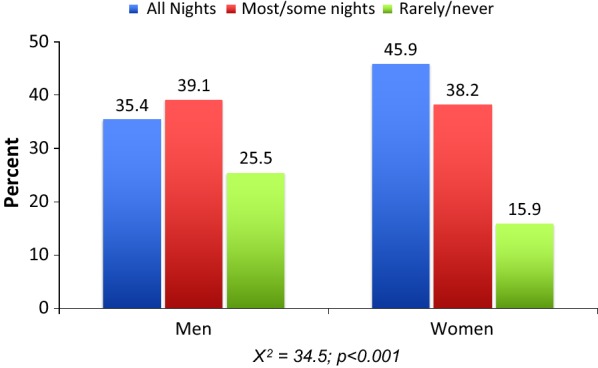
Variation in patterns of bed net use by gender. Akwa Ibom, Kebbi abd Nasarawa states, Nigeria; July–September, 2015

Almost all (94.4%) of the nets used by the study population were either LLINs or ITNs. Significant variations were evident in consistency of bed net use by sex (Fig. 1) and other background variables (Table 2). Specifically, women were significantly more likely than men to report consistent use of bed nets. Respondents aged between 18 and 24 years were more likely than others to report consistent use of bed nets, while those with no education were more likely to report the practice compared to their educated peers.

**Table 2:** Variations in patterns of bed net use by selected sociodemographic, ideational, household and community characteristics; respondents from households with at least one net

Background characteristics	Percent reporting specific pattern of bed net use			p-value
	Consistent use	Inconsistent use	Rarely or never	
Age				
18–24	53.60	33.53	12.87	< 0.001
25–34	42.01	39.75	18.24	
35–44	37.80	40.27	21.93	
45 +	37.93	38.46	23.61	
Respondent’s sex				
Male	35.43	39.06	25.52	< 0.001
Female	45.93	38.15	15.92	
Education				
None	58.71	32.50	9.79	< 0.001
Primary	36.65	42.20	21.15	
Secondary/higher	33.07	42.31	24.61	
Religion				
Christians	33.91	41.66	24.42	< 0.001
Muslim	54.41	34.72	10.87	
Traditional	54.47	24.53	20.75	
Exposure to malaria prevention messages on the media				
Not exposed	49.69	32.32	17.99	
Exposed	35.68	45.37	18.95	< 0.001
Perceived severity of malaria				
Lower	45.05	37.11	17.84	0.002
Higher	38.86	41.33	19.81	
Perceived susceptibility to malaria				
Lower	46.67	36.78	16.55	< 0.001
Higher	38.19	40.69	21.12	
Knew that malaria is caused by mosquito bite				
Did not know	41.65	29.05	29.31	< 0.001
Knew	43.45	39.43	17.22	
Perceived self-efficacy to prevent malaria				
Lower	35.30	38.17	26.52	< 0.001
Higher	46.49	38.48	15.03	
Perceived self-efficacy to detect a severe case of malaria				
Lower	38.55	37.53	23.92	< 0.001
Higher	44.98	38.72	16.30	
Perceived the response efficacy of bed nets				
No	43.09	39.09	17.82	
Yes	43.32	37.28	19.40	0.359
Perceived efficacy to use bed nets				
Lower	34.59	39.71	25.71	< 0.001
Higher	49.86	37.36	12.77	
Discussed malaria with someone				
No	52.12	24.49	23.39	< 0.001
Yes	41.72	40.65	17.63	
Discussed bed nets with someone in last 6 months				
Did not discuss	43.01	36.45	20.54	< 0.001
Discussed	43.56	42.80	13.65	
Score for positive attitudes towards bed nets				
Lower	43.41	36.30	20.29	< 0.001
Higher	42.89	40.98	16.13	
Perceived bed net use as the norm in the community				
Did not perceive	39.41	34.89	25.70	< 0.001
Perceived	45.61	40.65	13.73	
Knew of a place to purchase bed nets				
Did not know	38.15	39.96	21.90	< 0.001
Knew	57.38	33.96	8.66	
Willing to pay for bed nets				
No	37.87	37.31	24.82	< 0.001
Yes	48.55	39.48	11.97	
Community socioeconomic advantage				
Low	61.91	28.63	9.46	< 0.001
Medium	41.02	39.81	19.18	
High	29.81	45.10	25.09	
Household wealth				
Poorest	64.44	27.78	7.78	< 0.001
Poorer	54.80	31.83	13.36	
Middle	40.65	39.09	20.26	
Richer	31.86	43.84	24.30	
Richest	30.07	46.20	23.73	
State				
Akwa Ibom	30.61	40.75	28.64	< 0.001
Kebbi	66.72	25.93	7.35	
Nasarawa	32.71	50.49	16.80	
Place of residence				
Urban	39.31	41.72	18.97	
Rural	44.29	37.43	18.28	0.026

Results of bivariate analyses (n = 3884)

Respondents from Kebbi were more likely than their peers from Akwa Ibom or Nasarawa to report consistent use. Similarly, respondents from communities with low socioeconomic advantage were more likely to report consistent use than higher or medium socioeconomic advantage communities. With the exception of perceived response efficacy of bed nets, significant differences were shown in patterns of net use by ideational characteristics. Furthermore, the relationships of net use pattern with ideational variables were mostly positive. The only exceptions were the variables discussion of malaria and perceived susceptibility to malaria.

### Correlates of consistent use of bed nets

Table 3 presents results from the multilevel multinomial generalized structural equation model. The results show the likelihood (expressed as relative risk ratio) of consistent net use and the likelihood of inconsistent net use relative to using bed nets rarely or never. The table also show random effects—measured as variance—at the community level.

**Table 3:** Results of the multilevel multinomial regression of consistent use of bed nets on selected sociodemographic, ideational, household and community variables, Nigeria, 2015

Predictor	Using every night vs. using rarely/never		Using most or some nights vs. using rarely/never	
	Relative risk ratio	95% confidence interval	Relative risk ratio	95% confidence interval
Sociodemographic and media exposure variables				
Age in years	0.971***	0.957, 0.986	0.987^‡^	0.973, 1.002
Female gender (RC = male)	1.810***	1.385, 2.367	1.488**	1.156, 1.916
Education (RC = none)				
Primary	0.740^‡^	0.520, 1.054	0.690*	0.460, 0.972
Secondary and higher	0.670*	0.471, 0.953	0.638**	0.453, 0.898
Regularly listened to the radio (RC = no)	0.878	0.669, 1.153	0.873	0.671, 1.135
Regularly watched the television (RC = no)	0.562***	0.416, 0.759	0.757^‡^	0.569, 1.006
Religion (RC = christian)				
Muslim	0.442***	0.274, 0.711	0.741	0.473, 1.160
Others	0.290*	0.095, 0.886	0.541	0.182, 1.609
Exposed to malaria prevention message on the media in last 12 months (RC = not exposed)	1.665***	1.279, 2.168	1.939***	1.509, 2.492
Ideational characteristics				
Perceived severity of malaria (RC = did not perceive)	0.675**	0.529, 0.862	0.888	0.706, 1.117
Perceived susceptibility to malaria (RC = did not perceive)	0.936	0.748, 1.171	1.009	0.815, 1.249
Knew that malaria is caused by mosquito bite (RC = did not know)	1.342^‡^	0.950, 1.896	1.652**	1.182, 2.309
Reported perceived self-efficacy to prevent malaria (RC = did not report)	1.283^‡^	0.971, 1.695	1.048	0.807, 1.360
Reported perceived self-efficacy to detect severe malaria (RC = did not report)	1.040	0.792, 1.366	1.050	0.811, 1.360
Reported perceived response-efficacy of bed nets (RC = did not report)	1.468***	1.163, 1.854	0.897	0.719, 1.119
Reported perceived self-efficacy to use nets (RC = did not report)	1.802***	1.397, 2.324	1.340*	1.052, 1.706
Discussed malaria with others (RC = did not discuss)	1.450*	1.047,2.009	2.477***	1.775, 3.458
Discussed nets with others (RC = did not discuss)	1.382*	1.069, 1.787	1.283*	1.003, 1.640
Higher score for positive attitudes towards net use (RC = lower score)	1.547***	1.230, 1.944	1.551***	1.245, 1.929
Perceived net use to be the norm in the community (RC = did not perceive)	2.079***	1.657, 2.609	1.999***	1.608, 2.483
Knew where to buy nets in community	2.514***	1.841, 3.434	2.080***	1.528, 2.831
Willing to pay for nets (RC = not willing to pay)	1.655***	1.299, 2.109	1.824***	1.443, 2.307
Household/community variables				
Household size	0.862***	0.822, 0.904	0.904***	0.863, 0.946
Number of nets in household	1.867***	1.656, 2.104	1.491***	1.327, 1.675
Urban residence (RC = rural)	0.854	0.590, 1.237	0.772	0.539, 1.105
State of residence (RC = Akwa Ibom)				
Kebbi	17.328***	7.924, 37.893	4.107***	1.934, 8.721
Nasarawa	3.215***	1.861, 5.553	4.010***	2.379, 6.760
Household wealth quintile (RC = lowest)				
Second	0.767	0.484, 1.213	0.707	0.441, 1.132
Middle	0.591*	0.362, 0.964	0.720	0.439, 1.180
Fourth	0.545*	0.324, 0.918	0.832	0.495, 1.401
Highest	0.600^‡^	0.343, 1.049	0.979	0.563, 1.702
Community advantage index (RC = low)				
Medium	1.225	0.720, 2.084	0.944	0.563, 1.582
High	1.513	0.788, 2.905	1.444	0.770, 2.708
Random effects				
Cluster random effects/(SE)^a^	0.542*** (0.128)		0.480*** (0.121)	

*RC* reference category

^‡^p < 0.1; * p < 0.05; ** p < 0.01; *** p < 0.001

^a^Significance of community-level random effects is assessed using log-likelihood ratio tests (comparing models with and without random effects) with one-sided p-values because the null value is on the border of the parameter space

Among the sociodemographic and media variables assessed, only sex and exposure to malaria control messages on the media were strongly and positively associated with consistent and inconsistent use relative to using nets rarely/never. Compared to being a male, being a female significantly increased the chances of consistent bed net use by 81.0% (RRR = 1.810, CI = 1.385, 2.367) and that of using the net most or some nights by 48.8% (RRR = 1.488, CI = 1.156, 1.916) relative to never or rarely using. Respondents exposed to media messages on malaria control were 66.5% (RRR = 1.665, CI = 1.279, 2.168) more likely to report consistent net use and about 94% (RRR = 1.939, CI = 1.509, 2.492) more likely to report inconsistent use relative to rarely/never using. There was a negative dose–response with education: the likelihood of using nets every night and of using the net most or some nights compared to never or rarely using it decreased monotonically with level of education. In contrast, age, television viewing habit and non-Christian religion were negatively associated with consistent bed net use but made no significant difference for inconsistent use. There was a negative relationship with age such that one unit increase in age reduces the odds of using a net consistently by 3% (RRR = 0.971, CI = 0.957, 0.986) relative to never or rarely using nets. The likelihood of consistent bed net use was lower for Muslims and other non-Christians compared to Christians.

Many of the ideational variables were positively associated with both consistent and inconsistent use of bed nets rather than rarely or never using, including willingness to pay for nets, positive attitudes towards nets, perceived self-efficacy to use nets, and interpersonal communication about malaria. Other ideational variables that were positively associated with consistent and inconsistent net use rather than rarely/never using include descriptive norm about net use, interpersonal communication about nets and knowing a place to purchase nets in the community. For example, relative to using bed nets rarely or never, respondents who were willing to pay for nets had 65.5% (RRR = 1.655, CI = 1.299, 2.109) higher odds of reporting consistent use of bed net and 82.4% (RRR = 1.824, CI = 1.443, 2.307) higher odds of reporting inconsistent use compared to their peers who were not willing to pay for nets. In the same vein, the likelihood for consistent net use was 80.2% (RRR = 1.802, CI = 1.397, 2.324) higher and the relative risk ratio for inconsistent use was 34.0% (RRR = 1.340, CI = 1.052, 1.706) higher for respondents who perceived the self-efficacy to use nets. Awareness of mosquito bites as the cause of malaria was not strongly associated with consistent net use but significantly differentiated between using inconsistent use and using nets rarely or never (RRR = 1.652, CI = 1.182, 2.309). Conversely, perceived response-efficacy of bed nets significantly increased the likelihood of consistent use but made no difference for inconsistent use versus using rarely or never. Perceived severity of malaria was negatively correlated with consistent net use but made no difference for inconsistent use relative using nets rarely or never.

With the exception of community advantage index and urban residence, the household and community variables included in the model significantly predicted bed net use patterns. For example, one unit increase in household size reduced the likelihood of consistent net use by 13.8% and the likelihood of inconsistent use by about 10% relative to using rarely or never. Conversely, one unit increase in the number of nets in the household increased the likelihood of consistent use net by 86.7% and the likelihood of inconsistent use by 49%. Differences across states were considerable. Respondents from Kebbi and Nasarawa states were, respectively, more than 17-fold and more than threefold as likely to use consistently compared to those from Akwa Ibom. Household wealth was negatively associated with consistent bed net use but made no difference for inconsistent net use. Respondents from households in the middle or fourth wealth quintiles were 40.9 and 44.5% less likely, respectively, to use a bed net consistently compared to respondents from the poorest households. Finally, the community random effect was significant for consistent bed net use versus using rarely or never and for using bed nets most or some nights versus using rarely or never.

## Discussion

This study has examined the multilevel correlates of patterns of bed net use among men and women with under-5 children in Nigeria. Using household sample survey data collected in 2015, the study explored the roles of individual, household, and community variables. The focus on consistency of use of bed nets and assessment of the role of a wide array of ideational characteristics set this study apart from most other studies on the determinants of net use.

Consistent net use was not very common in the study population as only two-fifths of the respondents residing in households with nets reported sleeping under a bed net every night. The gap in ownership and consistent net use has been documented in other studies [9, 32]. The reasons for the gap are not entirely clear, although they may be related to factors that have been identified in literature for non-use of nets, including hot weather [33, 34], perceived low mosquito density [13, 33], poor net conditions [29, 35], insufficient number of nets in the household [35], and existence of netting on the windows of the dwelling structure [36]. The presence of window netting may explain why net use is lower for richer and more educated individuals. It is pertinent to note that while people in houses with window netting may feel protected from mosquitoes and perceive no need for bed nets, studies have demonstrated that window netting does not offer universal protection from mosquito bites indoors [37, 38].

The higher consistent use of bed nets among women compared to men is consistent with what prior studies have found in connection with sleeping under a bed net on the night before the study [15, 16, 18]. This finding might be due to sleeping arrangements within the household because women tend to share the same sleeping space with their young children. Contrary to what some studies have found regarding the relationship between education and use of bed nets [21, 22], this study revealed a negative relationship of education and bed net use. This finding is, however, consistent with what Russell et al. [23] found in another study in Nigeria.

Consistent with prior studies [10, 12, 21, 23, 28], findings from this study have underscored the important role of ideational characteristics in net use. The ideational characteristics that were positively and significantly associated with consistent net use included willingness to pay for nets, awareness of a place to obtain bed nets, perceived response efficacy of bed nets, discussion of malaria or bed nets with others, positive attitudes towards bed nets, perceived self-efficacy to use nets, and descriptive norms about bed net use. The strong association of positive attitude towards nets with consistent use makes intuitive sense and echoes evidence from the literature [39]. This study found a strong positive link between consistent net use and willingness to pay for nets. This finding is similar to studies that have found a connection between paying for nets and using them [21, 35]. The documented importance of perceived response-efficacy of bed nets in this study is consistent with what major theories of behaviour change—such as the Theory of Reasoned Action, and Ideation Model—have posited [40, 41] and what a few studies have found [27].

The negative relationship with perceived severity of malaria is counterintuitive and inconsistent with what some studies have found [12, 27]. While the reasons for this negative relationship are not entirely clear, literature on fear appeals provides a possible explanation. Scholars in the fear appeal tradition have argued that perceived severity of a disease will not necessarily lead to appropriate protective action in the absence of a commensurate level of perceived self-efficacy to take action and perceived response-efficacy of the prescribed solution [42, 43].

Finally, this study has provided additional evidence of the important role of unobserved community heterogeneity in individual use of bed nets. Indeed, the data indicate the presence of unmeasured (omitted) variables operating at the cluster level that are associated with the use of bed nets. In this respect, this study echoes what prior studies have found in connection with malaria prevention [12] and other health areas [44, 45]. While this study does not allow us to ascertain what these variables are, it is possible that they include factors related to community mobilization efforts, community engagement in malaria prevention efforts, housing density, and prevailing types of dwelling structures, such as the use or non-use of window netting.

The findings from this study have implications for programming. For example, the fact that not all households have nets indicates that net distribution through repeat mass campaigns and continuous channels continues to be relevant. The gap in ownership and consistent use observed in this study underscores the need for studies that use qualitative methods to assess the factors responsible for the gap. The finding that men were less likely than women to report use of bed net at any level (consistent or inconsistent) supports the relevance of a gender-sensitive approach. To be effective, the gender-sensitive approach should be informed by qualitative formative research designed to foster a better understanding of the reasons for low use of nets among men. The documented importance of ideational variables suggest that effective strategies for promoting consistent use of bed nets should adopt an approach based on the ideation framework. Efforts should focus on the ideational factors that are found in this study to be the greatest predictors of net use. Specifically, efforts to foster positive attitude towards bed nets, strengthen perceived response efficacy of bed nets and perceived self-efficacy to use nets, position net use as a community norm, promote discussion of malaria or bed nets with others, and increase malaria knowledge are relevant.

Furthermore, although net ownership was significantly higher in Akwa Ibom than in the other two states, consistent net use was significantly lower in this state than in the other states. It is pertinent to note that the three states differ considerably by the independent variables included in the estimated multilevel multinomial model and found to be associated with the consistency of bed net use. The finding that the three states differ significantly in terms of ideational variables has implications for state-specific health communication strategy for increasing the use of bed net in Nigeria. For example, the strategy for increasing bed net use in Akwa Ibom should emphasize perceived severity of malaria, strengthen the perceived self-efficacy for using nets, increase awareness about where to obtain nets, and promote willingness to pay for nets. In Kebbi, programmes should emphasize perceived severity of malaria, perceived susceptibility to malaria, positive attitudes towards net use, and perceived response-efficacy of nets. For Nasarawa, it is important to programmes to focus on increasing perceived severity of, and perceived susceptibility to malaria. Furthermore, considering the lower prevalence of regular radio listening and television viewing habits in Kebbi, it is important for programmes to explore other channels for reaching the population with relevant malaria prevention information. These additional channels can include mobile phones, community volunteers doing door-to-door activities, and community dialogues and events.

Finally, the finding that there are unmeasured variables at the community level affecting the consistency of net use underscores the importance of community-based approaches, including community dialogues, home visits, and working with community leaders.

This study has a few limitations that should be mentioned. First, the cross-sectional nature of the data precludes causal attribution. Second, the ideational variables and net use behaviour reported here are self-reported and might have been affected by social desirability bias. It is nonetheless pertinent to note that fieldworkers were trained to take appropriate steps to minimize this type of bias during data collection.

## Conclusion

The factors affecting consistent use of bed nets among respondents in Nigeria include socio-demographic, ideational, household and community factors. Efforts to promote consistent use of nets should adopt an approach based on the ideation framework with a particular focus on perceptions about severity, susceptibility, self-efficacy to use nets, and response-efficacy of bed net; awareness of place of purchase; willingness to pay for bed nets; attitudes towards net use; and descriptive norm about nets. Community-based strategies are also relevant.

## Abbreviations

HC3: Health Communication Capacity Collaborative
ITN: insecticide-treated net
LLIN: long-lasting insecticidal net
PMI: President’s Malaria Initiative
RBHS: Rebuilding Basic Health Services
USAID: United States Agency for International Development
WHO: World Health Organization
